# Placental FKBP51 mediates a link between second trimester maternal anxiety and birthweight in female infants

**DOI:** 10.1038/s41598-018-33357-3

**Published:** 2018-10-11

**Authors:** Katie L. Togher, Gerard W. O’Keeffe, Ali S. Khashan, Gerard Clarke, Louise C. Kenny

**Affiliations:** 1 The Irish Centre for Fetal and Neonatal Translational Research (INFANT), Cork University Maternity Hospital and University College Cork, Cork, Ireland; 2 APC Microbiome Institute, University College Cork, Cork, Ireland; 3 Department of Obstetrics and Gynaecology, Cork University Maternity Hospital, University College Cork, Cork, Ireland; 4 Department of Anatomy and Neuroscience, Western Gateway Building, University College Cork, Cork, Ireland; 5 School of Public Health, Western Gateway Building, University College Cork, Cork, Ireland; 6 Department of Psychiatry, Cork University Hospital and University College Cork, Cork, Ireland; 7 Faculty of Health and Life Sciences, University of Liverpool, Liverpool, United Kingdom

## Abstract

Prenatal distress is associated with adverse outcomes in affected offspring. Alterations in placental glucocorticoid signalling and subsequent foetal overexposure to glucocorticoids have been implicated as an underlying mechanism. Infant sex is emerging as an important factor in disease susceptibility. This study aimed to examine the effects of maternal distress across pregnancy on birth outcomes and placental glucocorticoid genes in a sex-dependent manner. Participants completed psychological distress questionnaires throughout pregnancy. Placental HSD11B2, NR3C1 and FKBP51 were analysed by real time PCR and cortisol was measured in new-born hair. Second trimester stress was negatively correlated with birthweight in males and positively correlated with placental NR3C1 mRNA in females. Second trimester anxiety was negatively correlated with birthweight and placental FKBP51 mRNA in females. In mediation analysis, placental FKBP51 mRNA expression was found to mediate the link between prenatal anxiety and birthweight. New-born cortisol was negatively correlated with second trimester anxiety and positively correlated with female placental FKBP51 mRNA levels. Again, FKBP51 mRNA was found to mediate the link between anxiety and new-born cortisol. These results highlight a role for FKBP51 in the placental response to prenatal distress in females. The precise role that placental FKBP51 has in foetal and infant development has not been extensively studied and warrants further investigations.

## Introduction

There is now a large body of evidence showing that the *in utero* experience is a critical determinant of future health^1–3^. One factor that has been extensively studied in this regard is the adverse effects of prenatal maternal psychological distress, which we define as the experience of significant levels of psychological stress, depression, and/or anxiety during pregnancy^4,5^. We have previously reported the incidence of this in pregnancy using the SCOPE (Screening for Pregnancy Endpoints) pregnancy cohort of nulliparous healthy pregnant women^6,7^. All participants completed a combination of validated questionnaires used to assess maternal psychological distress^5^. These included the 10-item Perceived Stress Scale (PSS) to measure psychological stress^8^, the 6-item version of State Trait Anxiety Inventory (STAI) to measure maternal anxiety^9^, and the Edinburgh Postnatal Depression Scale (EPDS) to measure maternal depressive symptoms in pregnancy^8,10^. We found that 15% of women experienced ‘very high levels of perceived psychological stress (≥90^th^ percentile score), 18% were classified as being as ‘very highly anxious’ (≥90^th^ percentile score), while 15% were classified as being ‘highly likely depressed’ (EPDS score >9)^5^. Collectively these data have shown that approximately one in seven women experience clinically significant levels of prenatal maternal psychological distress during pregnancy.

This is important as numerous epidemiological studies have reported that exposure to prenatal maternal psychological distress is a risk factor for a range of adverse short and long-term outcomes in affected offspring. These include an increased risk of adverse obstetric outcomes including caesarean delivery, preterm birth (PTB), low birth weight (LBW) and babies who are small for gestational age (SGA)^4,5,11–14^. Moreover prenatal maternal psychological distress has been proposed to be a risk factor for the development of immune^15,16^, metabolic^17,18^ and neuropsychiatric disorders^19–21^ later in life, with the relative risk varying by offspring sex^20,22–24^. These studies highlight the importance of prenatal maternal psychological distress as a risk factor for adverse outcomes in exposed offspring; however, the causal pathways mediating these associations are unclear.

The glucocorticoid hypothesis is the most widely studied biological mechanism proposed to mediate the association between prenatal maternal psychological distress and adverse outcomes^25^. During pregnancy, changes in the maternal hypothalamic-pituitary-adrenal (HPA) axis leads to an exponential rise in cortisol in the maternal circulation^26,27^. This cortisol stimulates the release of corticotrophin releasing hormone (CRH) from the placenta that enters the maternal circulation and further increases the production of cortisol forming a feed forward loop. As a result maternal cortisol levels are up to ten-fold higher than foetal levels^28^. This progressive increase in maternal cortisol is necessary for foetal organogenesis, however excessive foetal exposure may alter developmental trajectories^29^. The maternal-foetal cortisol gradient is maintained by the expression of 11β-hydroxysteroid dehydrogenase type 2 (HSD11B2) in the placental trophoblast which converts active cortisol into inactive cortisone^29^. Additionally, the glucocorticoid receptor (NR3C1) and FKPB51, a chaperone protein which regulates nuclear transport of NR3C1^30^, play an important role in the foetal response to cortisol. We and others have shown that maternal distress in late pregnancy reduces placental HSD11B2 expression^4,31,32^. We also found that the glucocorticoid receptor NR3C1 is upregulated by third trimester distress^4^. Increased methylation of placental FKBP51 has been reported following early third trimester stress^33^, however we previously observed no change in FKBP51 expression following distress in the third trimester^4^, indicating the need to examine other trimesters. Collectively these data suggest that prenatal maternal psychological distress may alter molecular mechanisms that regulate foetal exposure to maternal cortisol. Importantly alterations in the expression and regulation of HSD11B2, NR3C1 and/or FKBP51 has been linked to poor birth outcomes^34–36^ as well as neurobehavioral problems in infants^37–40^, suggesting that these may play a causal role in mediating the association between maternal distress and adverse outcomes.

In this study we sought to examine the relationships between psychological prenatal distress in the second and third trimester of pregnancy with birth outcomes and placental HSD11B2, NR3C1 and FKBP51 expression, as three key mediators of placental cortisol signalling. Moreover, we undertook causal mediation analysis to determine whether any changes in the placental expression of these genes were associated with birth outcomes using gender-sensitive methodology.

## Results

### Study Population

As part of a longitudinal cohort study at Cork University Maternity Hospital, 121 nulliparous pregnant women were recruited in their first or early second trimester of pregnancy. Placenta samples were available for 56 women. Detailed medical records were available for 55 of these women. 51 participants completed the PSS, STAI and EPDS in the second trimester (mean = 20.37 ± 0.85 gestational weeks (GW)). 46 participants completed these questionnaires in the third trimester (mean = 32.62 ± 1.03 GW) (Supplementary Fig. S1). New-born hair samples were available for 29 infants (51.8%). The mean ± SD PSS, STAI and EPDS scores were 14.41 ± 5.19, 4.87 ± 3.49 and 6.31 ± 4.16 in the second trimester and 12.13 ± 5.62, 5.27 ± 3.13 and 6.30 ± 4.63 in the third trimester respectively (Supplementary Fig. S2). Descriptive statistics for this cohort are presented in Tables 1 and 2.

**Table 1:** Descriptive statistics of continuous variables. Abbreviations: Standard Deviation (SD), Body Mass Index (BMI).

	Mean ± SD	Range	N
Maternal Age	30.80 ± 4.61	19–41	55
Maternal BMI	25.11 ± 4.15	19–39	55
Gestational Age (weeks)	39.75 ± 0.16	34–42	55
1 min Apgar Score	8.36 ± 0.18	3–10	55
5 min Apgar Score	9.51 ± 0.09	6–10	55
Birthweight	3623.45 ± 62.07	2170–4980	55
Birthweight Centiles	54.13 ± 3.48	9–100	55

**Table 2:** Descriptive statistics of categorical variables.

		Frequency (%)	N
Marital Status	*Single*	12.7	7
	*Married*	52.7	29
	*Defacto*	34.5	19
Employment	*Full-time*	87.3	48
	*Part-time*	5.5	3
	*Unemployed*	7.3	4
Country of Birth	*Ireland*	83.6	46
	*United Kingdom*	5.5	3
	*Poland*	5.5	3
	*Brazil*	1.8	1
	*Spain*	1.8	1
	*Romania*	1.8	1
Parity	*Nulliparous*	100	55
	*Multiparous*	0	0
Mode of Delivery	*Unassisted vaginal*	41.8	23
	*Operative vaginal*	36.4	20
	*Prelabour LSCS*	9.1	5
	*LSCS in labour*	12.7	7
Infant sex	*Male*	45.5	25
	*Female*	54.5	30
Gestational Age	*Term* (>*38 wks*)	98.2	54
	*Preterm* (<*37 wks*)	1.8	1
Gestational Size	*SGA*	1.8	1
	*AGA*	90.9	50
	*LGA*	7.3	4

Abbreviations: Lower Segment Caesarean Section (LSCS), SGA (Small for Gestational Age (SGA), Average for Gestational Age (AGA), Large for Gestational Age (LGA).

### Exposure to second trimester maternal anxiety negatively affects female birth weight

**Figure 1:**
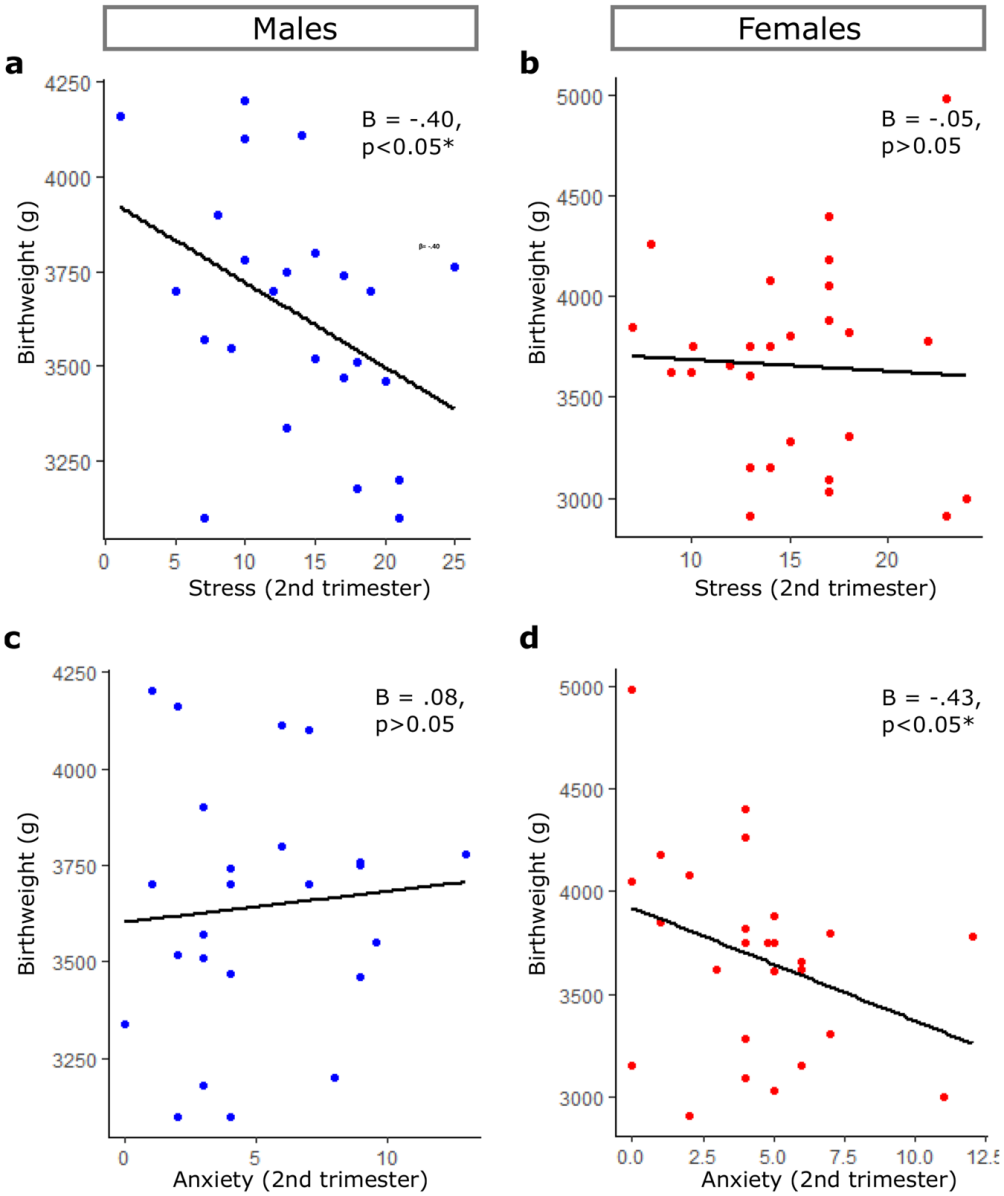
Second trimester distress correlates with reduced infant birthweight. Scatter plots of birthweight and (**a**,**b**) second trimester stress (PSS) and (**c**,**d**) second trimester anxiety (STAI) in males (blue) and females (red). Univariate linear regression analysis *p < 0.05.

We first sought to determine whether maternal psychological distress scores affected infant birth weight. To do this we examined the associations between PSS, STAI and EPDS scores in the second and/or third trimester with birth weight (mean = 3623 ± 460.3 g) (n = 55). We found that PSS scores in the second trimester were negatively correlated with male (p < 0.05), but not female birth weight (Fig. 1a,b). We found no associations between PSS scores measured in the third trimester or combined across pregnancy with male or female birth weight (Table 3). In contrast, second trimester STAI scores were negatively correlated with female (p < 0.05) but not male birth weight (Fig. 1c,d). We found no associations between STAI scores in the third trimester and birth weight in male or female infants (Table 3). When combined across pregnancy, STAI scores were negatively correlated with female birthweight (Table 3). We observed no association between EPDS scores in the second and/or third trimester with birth-weight of infants of either sex (Table 3). As infant birth weight was significantly altered by maternal BMI (Supplementary Table 1), we adjusted our regression model to examine the potential confounding effects of maternal BMI. When BMI was included in the analyses, the relationship between second trimester PSS scores and male birth weight disappeared (aβ = −0.32, t (23) = −1.72, p = 0.099). In contrast, second trimester anxiety remained correlated with female birth weight when BMI was included in the regression model (aβ = −0.46, t (26) = −2.71, p = 0.012). These data revealed a gender specific effect of maternal anxiety on birth weight in female infants.

**Table 3:** Maternal distress across pregnancy and birth outcomes.

	Both	Males	Females
**PSS (2** ^**nd**^ **trimester)**			
Birthweight	β = −0.18, t_50_ = −1.32, p = 0.19	**β = −0.40, t**_**23**_ ** = −2.08, p = 0.04**	β = −0.05, t_26_ = −0.25, p = 0.79
Birthweight Centiles	β = −0.18, t_50_ = −1.29, p = 0.20	β = −0.30, t23 = 1.50, p = 0.14	β = −0.16, t_26_ = −0.82, p = 0.41
**PSS (3** ^**rd**^ **trimester)**			
Birthweight	β = −0.10, t_45_ = −0.71, p = 0.47	β = −0.24, t_20_ = −1.08, p = 0.29	β = −0.05, t24 = −0.25, p = 0.80
Birthweight Centiles	β = −0.05, t_45_ = −0.37, p = 0.70	β = −0.15, t_20_ = −0.67, p = 0.50	β = 0.00, t_24_ = 0.01, p = 0.99
**PSS (Combined)**			
Birthweight	β = −0.266, t_41_ = −1.75, p = 0.09	β = −0.40, t_19_ = −1.87, p = 0.08	β = −0.22, t_21_ = −0.22, p = 0.34
Birthweight Centiles	β = −0.20, t_41_ = −1.331, p = 0.19	β = −0.29, t_19_ = −1.32, p = 0.20	β = −0.19, t_21_ = −0.86, p = 0.40
**STAI (2** ^**nd**^ **trimester)**			
Birthweight	*β* = −*0.25, t*_*50*_ = −*1.85, p* = *0.07*	β = 0.08, t_23_ = 0.39, p = 0.69	**β = −0.43, t**_**26**_ ** = −2.42, p = 0.02**
Birthweight Centiles	β = −0.19, t_50_ = −1.41, p = 0.16	β = −0.00, t_23_ = −0.02, p = 0.97	*β* = −*0.34, t*_*26*_ = −*1.80, p* = *0.08*
**STAI (3** ^**rd**^ **trimester)**			
Birthweight	*β* = −*0.28, t*_*45*_ = −*1.95, p* = *0.05*	β = −0.18, t_20_ = −0.81, p = 0.42	β = −0.33, t_24_ = −1.70, p = 0.10
Birthweight Centiles	β = −0.19, t_45_ = −1.33, p = 0.18	β = −0.19, t_20_ = −0.84, p = 0.40	β = −0.23, t_24_ = −1.17, p = 0.25
**STAI (Combined)**			
Birthweight	**β = −0.36, t**_**41**_ ** = −2.41, p = 0.02**	β = −0.05, t_19_ = −0.22, p = 0.83	**β = −0.50, t**_**21**_ ** = −2.59, p = 0.02**
Birthweight Centiles	β = −0.26, t_41_ = −1.73, p = 0.09	β = −0.15, t_19_ = −0.64, p = 0.53	β = −0.37, t_21_ = −1.79, p = 0.09
**EPDS (2** ^**nd**^ **trimester)**			
Birthweight	β = −0.13, t_50_ = −0.95, p = 0.34	β = −0.15, t_23_ = −0.72, p = 0.47	β = −0.13, t_26_ = −0.69, p = 0.49
Birthweight Centiles	β = −0.09, t_50_ = −0.69, p = 0.49	β = −0.22, t_23_ = −1.06, p = 0.29	β = −0.10, t_26_ = −0.51, p = 0.60
**EPDS (3** ^**rd**^ **trimester)**			
Birthweight	β = −0.12, t_45_ = −0.85, p = 0.39	β = −0.08, t_20_ = −0.37, p = 0.70	β = −0.14, t_24_ = −0.72, p = 0.47
Birthweight Centiles	β = −0.06, t_45_ = −0.39, p = 0.69	β = −0.08, t_20_ = −0.35, p = 0.73	β = −0.03, t_24_ = −0.15, p = 0.87
**EPDS (Combined)**			
Birthweight	β = −0.21, t_41_ = −1.37, p = 0.18	β = −0.12, t_19_ = −0.53, p = 0.61	β = −0.27, t_21_ = −1.26, p = 0.22
Birthweight Centiles	β = −0.11, t_41_ = −0.72, p = 0.48	β = −0.20, t_19_ = −0.87, p = 0.39	β = −0.12, t_21_ = −0.56, p = 0.58

Linear regression analysis. Data shown are crude unstandardized betas with corresponding t-statistic and p-values. Abbreviations: Perceived Stress Scale (PSS), State Trait Anxiety Inventory (STAI) and Edinburgh Postnatal Depression Scale (EPDS).

### Placental FKBP51 mediates the association between second trimester maternal anxiety and female birth weight

**Figure 2:**
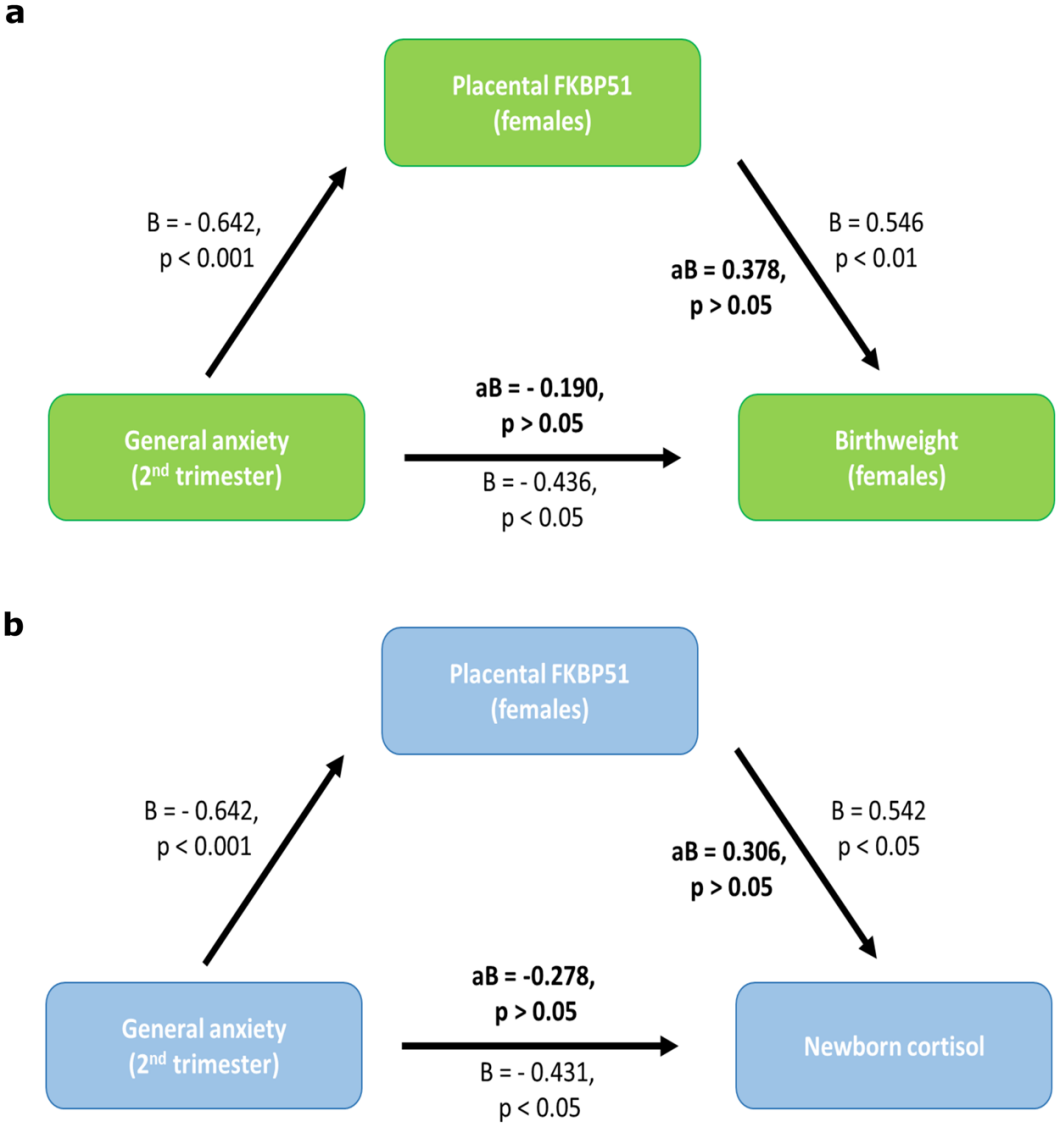
Placental FKBP51 mediates the relationship between prenatal anxiety and birthweight in females. Mediation Plots (**a**) Placental FKBP51 mediates the relationship between second trimester maternal anxiety and infant birthweight in females. (**b**) Placental FKBP51 mediates the relationship between second trimester maternal anxiety and new-born hair cortisol in females. Linear regression analysis.

**Figure 3:**
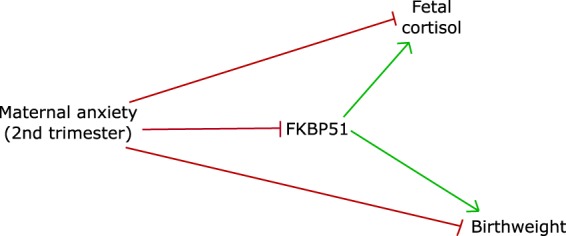
Summary Figure. Second trimester maternal anxiety decreases infant birthweight in female offspring by inhibiting placental FKBP51. Similarly second trimester maternal anxiety reduced foetal cortisol exposure by inhibiting FKBP51 in female offspring.

As second trimester anxiety was associated with female birth weight, we next examined the relationship between second trimester STAI scores and three key genes involved in glucocorticoid signalling in the placenta, HSD11B2, NR3C1 and FKBP51. In agreement with our findings on female birth weight (Fig. 1), we found a significant negative correlation between second trimester STAI scores and placental FKBP51 expression in females (β = −0.64, t (25) = −4.10, p < 0.0001), but not males (β = −0.53, t (23) = −1.78, p = 0.09). We found no significant associations between STAI scores in the second and/or third trimester and HSD11B2 or NR3C1 expression in males or females (Table 4). Additionally, no associations were observed between PSS and EPDS scores in the second or/and third trimester and placental expression of HSD11B2, NR3C1 and FKBP51 (Table 4), indicating that this effect is specific to heightened anxiety levels. We subsequently examined if placental FKBP51 expression was an independent predictor of infant birth weight. There was a significant association between placental FKBP51 with birth weight in female (β = 0.54, t (29) = 3.38, p = 0.002) but not male (β = −0.16, t (24) = −0.78, p = 0.44) infants (Table 5). These data show that second trimester anxiety (STAI scores) negatively correlates with both birth weight and placental FKBP51 in females, and that FKBP51 positively correlates with birth weight in females. Given these findings, we hypothesised that placental FKBP51 may be mediating the relationship between maternal anxiety and female birth weight. In support of this hypothesis when FKBP51 was included into the regression model the association between maternal anxiety and female birth weight was reduced (β = 0.19, t (25) = −0.816, p = 0.423) (Fig. 2a). These data show that the association between second trimester maternal anxiety and female birth weight is mediated by placental FKBP51 (Figs 2 and 3).

**Table 4:** Maternal distress across pregnancy and placental HSD11B2, NR3C1 and FKBP51 expression.

	Both	Males	Females
**PSS (2** ^**nd**^ **trimester)**			
*HSD11B2*	β = 0.26, t_49_ = 1.89, p = 0.07	β = 0.39, t_22_ = 1.99, p = 0.06	β = 0.09, t_26_ = 0.47, p = 0.64
*NR3C1*	β = −0.11, t_50_ = 0.80, p = 0.43	β = −0.15, t_23_ = −0.71, p = 0.49	**β = 0.42, t**_**26**_ ** = 2.33, p = 0.03**
*FKBP5*	β = −0.05, t_49_ = −0.32, p = 0.75	β = −0.04, t_23_ = −0.17, p = 0.87	β = −0.09, t_25_ = −0.42, p = 0.68
**PSS (3** ^**rd**^ **trimester)**			
*HSD11B2*	β = −0.11, t_45_ = −0.71, p = 0.48	β = −0.20, t_20_ = −0.90, p = 0.38	β = −0.02, t_24_ = −0.11, p = 0.92
*NR3C1*	β = −0.21, t_45_ = −1.39, p = 0.17	β = −0.31, t_20_ = −1.44, p = 0.17	β = −0.21, t_24_ = −0.59, p = 0.55
*FKBP5*	β = −0.16, t_44_ = −1.05, p = 0.29	β = −0.12, t_20_ = −0.53, p = 0.60	β = −0.21, t_23_ = −1.04, p = 0.32
**PSS (Combined)**			
*HSD11B2*	β = 0.13, t_41_ = 0.81, p = 0.42	β = 0.15, t_19_ = 0.64, p = 0.53	β = 0.08, t_21_ = 0.34, p = 0.74
*NR3C1*	β = −0.10, t_41_ = −0.65, p = 0.52	β = −0.39, t_19_ = −1.84, p = 0.08	β = 0.17, t_21_ = 0.81, p = 0.43
*FKBP5*	β = −0.14, t_40_ = −0.89, p = 0.38	β = −0.05, t_19_ = −0.23, p = 0.82	β = −0.28, t_20_ = −1.31, p = 0.20
**STAI (2** ^**nd**^ **trimester)**			
*HSD11B2*	β = −0.12, t_49_ = −0.81, p = 0.42	β = −0.12, t_22_ = −0.54, p = 0.59	β = −0.11, t_26_ = −0.56, p = 0.58
*NR3C1*	β = 0.15, t_50_ = 1.04, p = 0.31	β = 0.17, t_23_ = 0.78, p = 0.44	β = 0.13, t_26_ = 0.66, p = 0.52
*FKBP5*	**β = −0.46, t**_**49**_ ** = −3.59, p = 0.001**	β = −0.36, t_23_ = −1.79, p = 0.08	**β = −0.64, t**_**25**_ ** = −4.10, p = 0.000**
**STAI (3** ^**rd**^ **trimester)**			
*HSD11B2*	β = −0.00, t_45_ = −0.02, p = 0.98	β = −0.00, t_20_ = −0.03, p = 0.98	β = −0.01, t_24_ = −0.06, p = 0.95
*NR3C1*	β = −0.02, t_45_ = −0.14, p = 0.89	β = −0.01, t_20_ = −0.08, p = 0.94	β = −0.02, t_24_ = −0.09, p = 0.92
*FKBP5*	β = −0.21, t_44_ = −1.40, p = 0.17	β = −0.14, t_20_ = −0.61, p = 0.55	β = −0.31, t_23_ = −1.51, p = 0.15
**STAI (Combined)**			
*HSD11B2*	β = −0.04, t_41_ = −0.23, p = 0.82	β = −0.01, t_19_ = −0.05, p = 0.96	β = −0.06, t_21_ = −0.27, p = 0.79
*NR3C1*	β = 0.06, t_41_ = −0.40, p = 0.68	β = 0.06, t_19_ = 0.26, p = 0.79	β = 0.07, t_21_ = −0.30, p = 0.77
*FKBP5*	**β = −0.36, t**_**40**_ ** = −2.43, p = 0.019**	β = −0.26, t_19_ = −1.17, p = 0.26	β = −0.53, t_20_ = −2.70, p = 0.01
**EPDS (2** ^**nd**^ **trimester)**			
*HSD11B2*	β = 0.09, t_49_ = 0.67, p = 0.50	β = 0.06, t_22_ = 0.27, p = 0.79	β = 0.08, t_26_ = 0.39, p = 0.69
*NR3C1*	β = 0.03, t_50_ = 0.19, p = 0.85	β = −0.13, t_23_ = −0.63, p = 0.54	β = 0.16, t_26_ = 0.82, p = 0.42
*FKBP5*	β = −0.26, t_49_ = −1.83, p = 0.07	β = −0.24, t_23_ = −1.16, p = 0.26	β = −0.34, t_25_ = −1.74, p = 0.09
**EPDS (3** ^**rd**^ **trimester)**			
*HSD11B2*	β = −0.05, t_45_ = −0.34, p = 0.74	β = 0.08, t_20_ = 0.33, p = 0.75	β = −0.15, t_24_ = −0.72, p = 0.48
*NR3C1*	Β = −0.19, t_45_ = −1.25, p = 0.22	β = −0.24, t_20_ = −1.08, p = 0.29	Β = −0.16, t_24_ = −0.76, p = 0.46
*FKBP5*	β = −0.14, t_44_ = −0.92, p = 0.36	β = 0.07, t_20_ = 0.35, p = 0.73	β = −0.40, t_23_ = −2.09, p = 0.051
**EPDS (Combined)**			
*HSD11B2*	β = 0.06, t_41_ = 0.36, p = 0.72	β = 0.16, t_19_ = 0.67, p = 0.51	β = −0.06, t_21_ = −0.28, p = 0.78
*NR3C1*	Β = −0.12, t_41_ = −0.75, p = 0.46	β = −0.26, t_19_ = −1.17, p = 0.26	β = −0.00, t_21_ = −0.00, p = 0.99
*FKBP5*	β = −0.19, t_40_ = −1.27, p = 0.21	β = −0.02, t_19_ = −0.10, p = 0.92	**β = −0.44, t**_**20**_ ** = −2.16, p = 0.04**

Linear regression analysis. Data shown are crude unstandardized betas with corresponding t-statistic and p-values. Abbreviations: Perceived Stress Scale (PSS), State Trait Anxiety Inventory (STAI) and Edinburgh Postnatal Depression Scale (EPDS).

**Table 5:** Placental HSD11B2, NR3C1 and FKBP51 expression and neonatal outcomes.

	Both	Males	Females
**HSD11B2**			
Birthweight	*β* = 0.05, *t*_53_ = 0.36, *p* = 0.72	*β* = −0.17, *t*_23_ = −0.81, *p* = 0.42	*β* = 0.14, *t*_29_ = 0.76, *p* = 0.45
Birthweight Centiles	*β* = 0.14, *t*_53_ = 1.04, *p* = 0.30	*β* = −0.07, *t*_23_ = −0.36, *p* = 0.71	*β* = 0.21, *t*_29_ = 1.17, *p* = 0.24
**NR3C1**			
Birthweight	*β* = −0.07, *t*_54_ = −0.55, *p* = 0.58	*β* = −0.05, *t*_24_ = −0.24, *p* = 0.80	*β* = 0.09, *t*_29_ = −0.47, *p* = 0.63
Birthweight Centiles	*β* = −0.16, *t*_54_ = −1.24, *p* = 0.21	*β* = −0.03, *t*_24_ = −0.15, *p* = 0.87	*β* = −0.26, *t*_29_ = −1.43, *p* = 0.16
**FKBP51**			
Birthweight	*β* = 0.21, *t*_53_ = 1.58, *p* = 0.11	*β* = −0.16, *t*_24_ = −0.78, *p* = 0.44	***β* = 0.54**, ***t***_**29**_** = 3.38**, ***p* = 0.002**
Birthweight Centiles	*β* = 0.21, *t*_53_ = 1.57, *p* = 0.122	*β* = −0.09, _*t*24_ = −0.43, *p* = 0.67	***β* = 0.56**, ***t***_**29**_** = 3.51**, ***p* = 0.002**

Linear regression analysis. Data shown are crude unstandardized betas with corresponding t-statistic and p-values.

### Alterations in second trimester maternal anxiety and placental FKBP51 are associated with new-born cortisol levels

The placental and foetal response to glucocorticoids is crucial in determining foetal growth outcomes. This is highlighted by studies showing that exposure to synthetic glucocorticoids during pregnancy is associated with reductions in birth weight^41^, with some sex-specific outcomes also observed^42^. As FKBP51, negatively regulates nuclear transport of NR3C1^30^, we hypothesized that this may result in alterations in cortisol levels in infants. In an exploratory technique we measured cortisol levels in new-born hair as a potential retrospective measure of cortisol exposure *in utero*^43^. New-born hair samples were available for 29 infants in this cohort. We examined the relationship between maternal distress and infant cortisol levels. Maternal PSS or EPDS scores in the second and/or third trimester did not correlate with infant cortisol levels (Table 6). Surprisingly however, second trimester maternal anxiety (STAI) was negatively associated with infant hair cortisol levels (β = −0.43, t (25) = −2.33, p = 0.028). We next went on to examine the relationship between placental genes and birth outcomes with infant cortisol. Placental HSD11B2 (β = −0.02, t (28) = −0.10, p = 0.920) and NR3C1 (β = −0.19, t (28) = −1.00, p = 0.325) were not related to new-born cortisol. Intriguingly however, FKBP51 expression was positively correlated with infant cortisol levels in females only (β = 0.54, t (13) = 2.23, p = 0.045). As both maternal anxiety and FKBP51 were related to new-born cortisol levels, when we included both in a mediation model, the relationship between second trimester anxiety (aβ = −0.27, t (11) = −0.62, p = 0.550) and FKBP51 (aβ = 0.30, t (11) = 0.68, p = 0.510) with new-born cortisol levels was reduced. These data suggest that FKBP51 mediates the relationship between second trimester anxiety and new-born cortisol levels (Fig. 2b). As the relationship between second trimester anxiety and infant birthweight and cortisol levels were both independently mediated by placental FKBP51, we hypothesized that foetal cortisol may be the biological mediator behind these associations. However, there was no correlation observed between new-born cortisol levels and infant birth weight, suggesting the biological mediators linking placental FKBP51 with infant birthweight may not be related to foetal cortisol exposure.

**Table 6:** Maternal distress across pregnancy and cortisol levels in new-born hair.

	Both	Males	Females
**New-born Cortisol**			
PSS (2^nd^ trimester)	β = −0.13, t_25_ = −0.68, p = 0.49	β = 0.12, t_13_ = 0.42, p = 0.68	β = −0.41, t_25_ = −1.44, p = 0.17
PSS (3^rd^ trimester)	β = −0.10, t_26_ = −0.53, p = 0.59	β = 0.01, t_13_ = 0.03, p = 0.97	β = −0.17, t_12_ = −0.60, p = 0.55
PSS (Combined)	β = −0.22, t_23_ = −1.03, p = 0.31	β = 0.15, t_12_ = 0.52, p = 0.61	**−β = −0.63, t**_**10**_ ** = −2.45, p = 0.04**
STAI (2^nd^ trimester)	**β = −0.43, t**_**25**_ ** = −2.33, p = 0.03**	*β* = −*0.51, t*_*13*_ = −*2.06, p* = *0.06*	*β* = −*0.51, t*_*11*_ = −*1.91, p* = *0.08*
STAI (3^rd^ trimester)	β = −0.16, t_26_ = −0.81, p = 0.42	β = −0.23, t_13_ = −0.83, p = 0.42	β = −0.10, t_12_ = −0.36, p = 0.72
STAI (Combined)	*β* = −*0.38, t*_*23*_ = −*1.90, p* = *0.07*	β = −0.42, t_12_ = −1.51, p = 0.16	β = −0.43, t_10_ = −1.43, p = 0.188
EPDS (2^nd^ trimester)	*β* = −*0.36, t*_*25*_ = −*1.91, p* = *0.067*	β = −0.15, t_13_ = −0.54, p = 0.59	β = −0.42, t_11_ = −1.46, p = 0.17
EPDS (3^rd^ trimester)	β = −0.15, t_26_ = −0.80, p = 0.42	β = −0.04, t_13_ = −0.15, p = 0.87	β = −0.24, t_12_ = −0.84, p = 0.41
EPDS (Combined)	β = −0.23, t_23_ = −1.11, p = 0.28	β = −0.02, t_12_ = −0.08, p = 0.94	β = −0.36, t_10_ = −1.15, p = 0.28

Linear regression analysis. Data shown are crude unstandardized betas with corresponding t-statistic and p-values. Abbreviations: Perceived Stress Scale (PSS), State Trait Anxiety Inventory (STAI) and Edinburgh Postnatal Depression Scale (EPDS).

## Discussion

A large proportion of women report experiencing psychological distress throughout their pregnancy^5,32^. This is important as prenatal distress has been linked to a wide range of poor obstetric and neonatal outcomes as well as an increased risk of disease in childhood and adulthood for exposed offspring. Of particular importance prenatal distress is commonly linked to birthweight and birth size^44^. Reduced birthweight remains a significant clinical challenge as it is often associated with increased mortality and morbidity^45^. Additionally, infants of lower birthweights are at an increased risk of developmental impairments in childhood, particularity in relation to neurodevelopment^46,47^. Whilst the poor outcomes associated with being born low birthweight are well documented, the prenatal determinants linking psychological distress and birthweight are not very well understood.

Here we find the effect of maternal distress on birth outcomes to depend on the type of distress, timing of distress and sex of the infant. We initially observed a significant relationship between second trimester stress and reduced infant birthweight in males. However, this association disappeared after adjustment for maternal BMI. Most notably we observe a significant negative correlation between birthweight and second trimester anxiety, consistent with a recent report^48^. When stratified based on sex, this relationship was only observed in females. This sex difference pertaining to birthweight and prenatal anxiety has previously been reported where males born from anxious pregnancies had increased birthweight compared to male controls, and females born from anxious mothers had reduced birthweights compared to female controls^49^. Anxious mothers of females are more likely to develop obstetric complications, whereas anxious mothers of males are not^50^. Male fetuses are generally more vulnerable to the effects of maternal distress^51^. It has been postulated that under conditions of adversities the male fetus favors growth at the expense of other developmental processes, whereas the female fetus conserves growth, thus being born at lower weights but with fewer morbidities in later life^50^. In support of this, mid pregnancy exposure to dexamethasone, a synthetic glucocorticoid, was found to decrease maternal blood in sinusoids of the female but not male placenta, restricted blood flow may mechanistically explain restricted growth in female fetuses^52^.

At a biological level, sex specific responses in the placenta to maternal perturbations may explain why one sex is more vulnerable over the other^53^. Sex-specific responses to maternal glucocorticoids, or more specifically how the placenta regulates glucocorticoids differentially may play a role^54,55^. In this study we focused on three genes in the placenta involved in glucocorticoid regulation; HSD11B2, NR3C1 and FKBP51. Inconsistent with our previous work^4^ and work of others^31^, we do not observe a reduction in HSD11B2 following prenatal distress, however the mean stress score in this population was relatively low and the effect of maternal stress on HSD11B2 expression has been shown to be dependent on severity^56^. Second trimester maternal stress increased NR3C1 expression in female placentae. This increase in NR3C1 we observe among females but not males could again represent an adaptive response of the female placenta in response to maternal distress.

FKBP51 is a chaperone protein that interacts with steroid hormone receptors through heat shock protein 90 (HsP90), inhibiting the activation of the glucocorticoid receptor and the progesterone receptor (PR) and increasing the activation of the androgen receptor^57^. Consistent with previous reports^33,58^ we find prenatal distress to reduce placental FKBP51 expression. Importantly we find placental FKBP51 to mediate a relationship between second trimester anxiety and infant birth weight in females only, suggesting a critical role for this chaperone protein in the female foetal response to maternal anxiety. As FKBP51 regulates the glucocorticoid receptor we hypothesised that placental changes in FKBP51 would result in alterations in foetal cortisol. Indeed, we observed a positive correlation between placental FKBP51 and new-born cortisol levels in female infants. Of interest, second trimester anxiety was the only distress variable that influenced new-born cortisol levels. Following our mediation analysis, we showed second trimester maternal anxiety decreases new-born cortisol levels by reducing FKBP51 in female placentae (Fig. 3). However, no association between new-born cortisol levels and birthweight were found. Alternatively, the inhibitory action of FKBP51 on the PR may underlie the relationship between maternal anxiety and infant birthweight. Progesterone supplementation is commonly administered to women at risk of preterm birth and women who receive progesterone are less likely to deliver a preterm or deliver low birthweight infants^59^. As the relationship we observed between placental FKBP51 and birthweight was specific to females, it is of particular interest that increased maternal serum placental progesterone in the first trimester has been found to be associated with increased birthweight in females, with no significant effect on males^60^. Therefore, it may be possible that the inhibitory actions of FKBP51 on the PR may underlie the link between maternal anxiety and female birthweight. Future studies into this relationship should help to elucidate these mechanisms. None the less our finding, together with the previously reported relationship between placental FKBP51 methylation and neurobehavioral problems in infants^40^, suggests placental FKBP51 as a novel player in foetal programming.

Identifying vulnerable periods of development where the foetus (and placenta) are most susceptible to environmental perturbations is a growing area of research. By prospectively examining women in the second and third trimester we have been able to identify the second trimester as a critical window where the foetus might be most susceptible to the effects of maternal anxiety. This is consistent with a number of similar studies that have demonstrated alterations in the mRNA expression and/or methylation levels of glucocorticoid regulating genes, following second trimester maternal distress^31,33,61^. Similarly, mid-gestation exposure to severe life events, particularly in months 5 and 6 of pregnancy have been shown to heighten the risk of adverse neonatal outcomes^62^. The vulnerability of the second trimester is further evident by a number of studies that have shown second trimester stress to predict poor neurodevelopment in infants^63–66^. The second trimester is a period of rapid foetal growth, particularly for the foetal brain^67^. Further, the foetal HPA response becomes active from 20 weeks of pregnancy^68^, therefore maternal stress arising in this period may have a more detrimental impact on development.

The current study has several strengths and limitations. Although the sample size used in this study is comparable to that of previously published work examining maternal distress and placental gene expression^31,32^, we acknowledge the small sample size and suggest this research be carried out on a larger scale, although appreciate the difficulties in running large scale placental collection studies for mRNA analysis^69^. Due to the limited sample size in this cohort, we did not routinely adjust for potential confounders outside of maternal age and BMI, therefore it may be possible that maternal lifestyle factors and socioeconomic status may be further influencing this relationship. We report significant associations with prenatal distress and birthweight we would like to highlight that only one (1.8%) infant in this cohort was born <2500 g, the WHO estimate for clinically defined low birthweight^70^. Validating this work in a cohort of clinically defined low birthweight infants will be an important next step in unravelling the role of FKBP51 in the foetal response to maternal anxiety. By prospectively examining maternal distress in the second and third trimester of pregnancy we have been able to identify the second trimester as a crucial period during development whereby the foetus may be most susceptible to the effects of maternal distress. This will add to the growing body of literature examining critical windows of foetal development.

Overall this study is important as it identifies a crucial role for the timing of distress, the type of distress and foetal sex in the relationship between prenatal distress and placental gene expression. This adds to the existing literature supporting a role for alterations in placental glucocorticoid signalling following prenatal distress. To our knowledge this is the first study to identify an association between placental FKBP51 and infant birthweight. Importantly we identify this gene to be a key mediator underlying a relationship between prenatal anxiety and birthweight in females, which highlights the crucial role placental signalling has in terms of exposure to maternal distress and infant development. The identification of this relationship warrants further investigation into the precise role that FKBP51 has in foetal development.

## Methods

### Participants

This study received full ethical approval from the Clinical Research Ethics Committee of Cork Teaching Hospitals and was carried out in accordance with the guidelines and regulations outlined in the ethics. Nulliparous pregnant women enrolled in the IMPROvED study^71^ at Cork University Maternity Hospital were invited to participate in this study. After giving informed consent, participants completed the PSS, STAI, and EPDS in the second and/or third trimesters of pregnancy. Detailed demographic and medical information was acquired from the participants’ medical records.

### New-born Hair Collection and Processing

New-born hair was acquired from the posterior vortex of the new-borns head within 24 h of birth and stored at room temperature until processing. 1 mg of hair was incubated in 1 ml of methanol at 50 °C for 24 h. Samples were sonicated for 30 min at 37 °C followed by another incubation for 24 h at 50 °C. The supernatant was removed and evaporated under nitrogen and the pellet was resuspended in Phosphate Buffered Solution. Cortisol concentration was determined by ELISA as per the manufacturer’s instructions (Enzo life Sciences).

### Placental collection and real-time PCR

Placenta biopsies were collected from 56 participants within 2 h of delivery, washed in dH_2_O and immediately stored at −80 °C. RNA was extracted from placental samples using Trizol reagent as previously described^4^. Briefly, placental samples were homogenised in Trizol and left on ice for 10 min. Samples were centrifuged and the supernatant was incubated in chloroform at room temperature for 5 min followed by centrifugation for 15 min at 4 °C to remove the aqueous phase. RNA was isolated by incubation of the aqueous phase with propanol at room temperature for 10 min. Samples were centrifuged and the pellet washed in 70% ethanol before resuspension in RNAse free H_2_O (Sigma). RNA quality and quantity were determined by the Nanodrop 1000. RNA was reverse transcribed into cDNA (400 ng/ml) using the high capacity cDNA reverse transcription kit (Applied Biosystems) under the following parameters: 25 °C for 10 min, 37 °C for 120 min, 85 °C for 5 min and 4 °C for at least 10 min. Real time PCR was performed with the following targets; GAPDH, HSD11B2, NR3C1 and FKBP51 (Integrated DNA Technologies; IDT) using the following parameters; 50 °C for 2 min, 95 °C for 10 min, 50 repetitions of 95 °C for 15 s and annealing/elongating at 60 °C, as previously described^4^. All samples were run in triplicate and gene expression was determined using the 2−ΔΔcycle threshold (2dCT) method^72^ with GAPDH as the reference.

### Statistical Analysis

Data analysis was performed on SPSS v22. Scatterplots were produced using R v3.4.2, library ggplot2. Normality of predictor and outcome variables were tested for using Kolmogorov-Smirnov tests. Questionnaire scores, birthweight and birthweight centiles were normally distributed. Placental gene expression and hair cortisol levels displayed a non-normal distribution and were log transformed prior to analysis (Supplementary Fig. S3). A cumulative stress, depression and anxiety score was determined for each participant by summing relevant questionnaire scores from the second and third trimester. This cumulative score is referred to as PSS, STAI or EPDS *combined* throughout the results section. Outliers were determined using a Grubbs test and removed if p < 0.05. Relationships were determined using linear regression analysis. Due to the limited sample size in this cohort, regression models were adjusted for maternal age and BMI, only when these demographics correlated with the predictor and/or outcome variables (p < 0.05) (Supplementary Table S1).

## Electronic supplementary material

Supplementary Information

## Data Availability

The raw data used to complete this work is available to readers in Supplementary Information, Supplementary Table S3.

## References

[1] Langley-Evans SC (2006). Developmental programming of health and disease. Proceedings of the Nutrition Society.

[2] Cao-Lei L, Laplante DP, King S (2016). Prenatal Maternal Stress and Epigenetics: Review of the Human Research. Current Molecular Biology Reports.

[3] Todd N, Valleron AJ, Bougneres P (2017). Prenatal loss of father during World War One is predictive of a reduced lifespan in adulthood. Proc Natl Acad Sci USA.

[4] Togher KL, Treacy E, O’Keeffe GW, Kenny LC (2017). Maternal distress in late pregnancy alters obstetric outcomes and the expression of genes important for placental glucocorticoid signalling. Psychiatry research.

[5] Khashan A. S., Everard C., McCowan L. M. E., Dekker G., Moss-Morris R., Baker P. N., Poston L., Walker J. J., Kenny L. C. (2014). Second-trimester maternal distress increases the risk of small for gestational age. Psychological Medicine.

[6] Kenny LC (2014). Early pregnancy prediction of preeclampsia in nulliparous women, combining clinical risk and biomarkers: the Screening for Pregnancy Endpoints (SCOPE) international cohort study. Hypertension.

[7] Larsen PS (2013). Pregnancy and birth cohort resources in europe: a large opportunity for aetiological child health research. Paediatric and perinatal epidemiology.

[8] Cohen S, Kamarck T, Mermelstein R (1983). A global measure of perceived stress. Journal of health and social behavior.

[9] Marteau TM, Bekker H (1992). The development of a six-item short-form of the state scale of the Spielberger State-Trait Anxiety Inventory (STAI). Br J Clin Psychol.

[10] Rubertsson, C., Borjesson, K., Berglund, A., Josefsson, A. & Sydsjo, G. The Swedish validation of Edinburgh Postnatal Depression Scale (EPDS) during pregnancy. *Nord J Psychiatr*y **65**, 414–418, Epub 2011 Jul 5, 10.3109/08039488.2011.59060610.3109/08039488.2011.590606 (2011). 10.3109/08039488.2011.590606 21728782

[11] Liou, S. R., Wang, P. & Cheng, C. Y. Effects of prenatal maternal mental distress on birth outcomes. *Women and birth: journal of the Australian College of Midwives*, 10.1016/j.wombi.2016.03.00410.1016/j.wombi.2016.03.004. (2016). 10.1016/j.wombi.2016.03.004 27079210

[12] Grote NK (2010). A meta-analysis of depression during pregnancy and the risk of preterm birth, low birth weight, and intrauterine growth restriction. Archives of general psychiatry.

[13] Ding X-X (2014). Maternal anxiety during pregnancy and adverse birth outcomes: A systematic review and meta-analysis of prospective cohort studies. Journal of affective disorders.

[14] Rose MS, Pana G, Premji S (2016). Prenatal Maternal Anxiety as a Risk Factor for Preterm Birth and the Effects of Heterogeneity on This Relationship: A Systematic Review and Meta-Analysis. BioMed research international.

[15] Flanigan C, Sheikh A, Nwaru BI (2016). Prenatal maternal psychosocial stress and risk of asthma and allergy in their offspring: protocol for a systematic review and meta-analysis. NPJ Prim Care Respir Med.

[16] Khashan AS (2012). Prenatal stress and risk of asthma hospitalization in the offspring: a Swedish population-based study. Psychosomatic medicine.

[17] Entringer S (2013). Impact of stress and stress physiology during pregnancy on child metabolic function and obesity risk. Curr Opin Clin Nutr Metab Care.

[18] Entringer S (2008). Prenatal psychosocial stress exposure is associated with insulin resistance in young adults. American journal of obstetrics and gynecology.

[19] Class QA (2014). Offspring psychopathology following preconception, prenatal and postnatal maternal bereavement stress. Psychological medicine.

[20] Khashan AS (2011). Risk of affective disorders following prenatal exposure to severe life events: a Danish population-based cohort study. Journal of psychiatric research.

[21] Khashan AS (2008). Higher risk of offspring schizophrenia following antenatal maternal exposure to severe adverse life events. Archives of general psychiatry.

[22] Quarini C (2016). Are female children more vulnerable to the long-term effects of maternal depression during pregnancy?. Journal of affective disorders.

[23] Mueller BR, Bale TL (2008). Sex-specific programming of offspring emotionality after stress early in pregnancy. The Journal of neuroscience: the official journal of the Society for Neuroscience.

[24] Weinstock M (2007). Gender Differences in the Effects of Prenatal Stress on Brain Development and Behaviour. Neurochemical research.

[25] Reynolds RM (2013). Glucocorticoid excess and the developmental origins of disease: two decades of testing the hypothesis–2012 Curt Richter Award Winner. Psychoneuroendocrinology.

[26] Nolten WE, Lindheimer MD, Rueckert PA, Oparil S, Ehrlich EN (1980). Diurnal patterns and regulation of cortisol secretion in pregnancy. The Journal of clinical endocrinology and metabolism.

[27] Goland RS, Wardlaw SL, Blum M, Tropper PJ, Stark RI (1988). Biologically active corticotropin-releasing hormone in maternal and fetal plasma during pregnancy. American journal of obstetrics and gynecology.

[28] Riley SC, Challis JR (1991). Corticotrophin-releasing hormone production by the placenta and fetal membranes. Placenta.

[29] Togher Katie L, Togher Katie L, O'Keeffe Majella M, O'Keeffe Majella M, Khashan Ali S, Khashan Ali S, Gutierrez Humberto, Gutierrez Humberto, Kenny Louise C, Kenny Louise C, O'Keeffe Gerard W, O'Keeffe Gerard W (2014). Epigenetic regulation of the placental HSD11B2 barrier and its role as a critical regulator of fetal development. Epigenetics.

[30] Zhang X, Clark AF, Yorio T (2008). FK506-binding protein 51 regulates nuclear transport of the glucocorticoid receptor beta and glucocorticoid responsiveness. Investigative ophthalmology & visual science.

[31] Seth S, Lewis AJ, Saffery R, Lappas M, Galbally M (2015). Maternal Prenatal Mental Health and Placental 11beta-HSD2 Gene Expression: Initial Findings from the Mercy Pregnancy and Emotional Wellbeing Study. International journal of molecular sciences.

[32] O’Donnell KJ (2012). Maternal prenatal anxiety and downregulation of placental 11beta-HSD2. Psychoneuroendocrinology.

[33] Monk C (2016). Distress During Pregnancy: Epigenetic Regulation of Placenta Glucocorticoid-Related Genes and Fetal Neurobehavior. The American journal of psychiatry.

[34] Dy J, Guan H, Sampath-Kumar R, Richardson BS, Yang K (2008). Placental 11beta-hydroxysteroid dehydrogenase type 2 is reduced in pregnancies complicated with idiopathic intrauterine growth Restriction: evidence that this is associated with an attenuated ratio of cortisone to cortisol in the umbilical artery. Placenta.

[35] Causevic M, Mohaupt M (2007). 11beta-Hydroxysteroid dehydrogenase type 2 in pregnancy and preeclampsia. Molecular aspects of medicine.

[36] Filiberto AC (2011). Birthweight is associated with DNA promoter methylation of the glucocorticoid receptor in human placenta. Epigenetics.

[37] Conradt E, Lester BM, Appleton AA, Armstrong DA, Marsit CJ (2013). The roles of DNA methylation of NR3C1 and 11beta-HSD2 and exposure to maternal mood disorder in utero on newborn neurobehavior. Epigenetics.

[38] Appleton AA, Lester BM, Armstrong DA, Lesseur C, Marsit CJ (2015). Examining the joint contribution of placental NR3C1 and HSD11B2 methylation for infant neurobehavior. Psychoneuroendocrinology.

[39] Marsit CJ, Maccani MA, Padbury JF, Lester BM (2012). Placental 11-beta hydroxysteroid dehydrogenase methylation is associated with newborn growth and a measure of neurobehavioral outcome. PloS one.

[40] Paquette AG (2014). Placental FKBP5 genetic and epigenetic variation is associated with infant neurobehavioral outcomes in the RICHS cohort. PloS one.

[41] Khan AA (2011). Does in utero exposure to synthetic glucocorticoids influence birthweight, head circumference and birth length? A systematic review of current evidence in humans. Paediatric and perinatal epidemiology.

[42] Stevenson D (2000). Sex differences in outcomes of very low birthweight. Archives of Disease in Childhood. Fetal and Neonatal Edition.

[43] Hollanders JJ (2017). Interpretation of glucocorticoids in neonatal hair: a reflection of intrauterine glucocorticoid regulation?. Endocrine connections.

[44] Bussières E-L (2015). Maternal prenatal stress and infant birth weight and gestational age: A meta-analysis of prospective studies. Developmental Review.

[45] Jeschke E (2016). Mortality and Major Morbidity of Very-Low-Birth-Weight Infants in Germany 2008–2012: A Report Based on Administrative Data. Frontiers in Pediatrics.

[46] Walhovd KB (2012). Long-term influence of normal variation in neonatal characteristics on human brain development. Proceedings of the National Academy of Sciences of the United States of America.

[47] Howe T-H, Sheu C-F, Hsu Y-W, Wang T-N, Wang L-W (2016). Predicting neurodevelopmental outcomes at preschool age for children with very low birth weight. Research in Developmental Disabilities.

[48] Pinto Tiago Miguel, Caldas Filipa, Nogueira-Silva Cristina, Figueiredo Bárbara (2017). Maternal depression and anxiety and fetal-neonatal growth. Jornal de Pediatria.

[49] Kaitz M, Mankuta D, Rokem AM, Faraone SV (2015). Relation between maternal antenatal anxiety and infants’ weight depends on infants’ sex: A longitudinal study from late gestation to 1-month post birth. Journal of psychosomatic research.

[50] Kaitz M, Mankuta D, Rokem AM, Faraone SV (2014). Moderate antenatal anxiety symptoms and birth outcomes of boys and girls. Journal of psychosomatic obstetrics and gynaecology.

[51] Stormer C (2011). Sex differences in the consequences of early-life exposure to epidemiological stress–a life-history approach. American journal of human biology: the official journal of the Human Biology Council.

[52] O’Connell BA (2017). The Placental Response to Excess Maternal Glucocorticoid Exposure Differs Between the Male and Female Conceptus in Spiny Mice. Biology of Reproduction.

[53] Clifton VL (2010). Review: Sex and the Human Placenta: Mediating Differential Strategies of Fetal Growth and Survival. Placenta.

[54] Hodyl NA (2010). Sex-specific associations between cortisol and birth weight in pregnancies complicated by asthma are not due to differential glucocorticoid receptor expression. Thorax.

[55] Cuffe James S M, Saif Zarqa, Perkins Anthony V, Moritz Karen M, Clifton Vicki L (2017). Dexamethasone and sex regulate placental glucocorticoid receptor isoforms in mice. Journal of Endocrinology.

[56] Welberg LA, Thrivikraman KV, Plotsky PM (2005). Chronic maternal stress inhibits the capacity to up-regulate placental 11beta-hydroxysteroid dehydrogenase type 2 activity. The Journal of endocrinology.

[57] Stechschulte LA, Sanchez ER (2011). FKBP51 – a selective modulator of glucocorticoid and androgen sensitivity. Curr Opin Pharmacol.

[58] Kertes DA (2016). Prenatal Maternal Stress Predicts Methylation of Genes Regulating the Hypothalamic-Pituitary-Adrenocortical System in Mothers and Newborns in the Democratic Republic of Congo. Child development.

[59] Dodd JM, Crowther CA, Cincotta R, Flenady V, Robinson JS (2005). Progesterone supplementation for preventing preterm birth: a systematic review and meta-analysis. Acta obstetricia et gynecologica Scandinavica.

[60] Hartwig IRV, Pincus MK, Diemert A, Hecher K, Arck PC (2013). Sex-specific effect of first-trimester maternal progesterone on birthweight. Human Reproduction.

[61] Murgatroyd C, Quinn JP, Sharp HM, Pickles A, Hill J (2015). Effects of prenatal and postnatal depression, and maternal stroking, at the glucocorticoid receptor gene. Translational psychiatry.

[62] Class QA, Lichtenstein P, Langstrom N, D’Onofrio BM (2011). Timing of prenatal maternal exposure to severe life events and adverse pregnancy outcomes: a population study of 2.6 million pregnancies. Psychosomatic medicine.

[63] King S, Laplante DP (2005). The effects of prenatal maternal stress on children’s cognitive development: Project Ice Storm. Stress.

[64] Glynn LM, Wadhwa PD, Dunkel-Schetter C, Chicz-Demet A, Sandman CA (2001). When stress happens matters: effects of earthquake timing on stress responsivity in pregnancy. American journal of obstetrics and gynecology.

[65] Buss C, Davis EP, Muftuler LT, Head K, Sandman CA (2010). High pregnancy anxiety during mid-gestation is associated with decreased gray matter density in 6-9-year-old children. Psychoneuroendocrinology.

[66] Buss C, Davis EP, Hobel CJ, Sandman CA (2011). Maternal pregnancy-specific anxiety is associated with child executive function at 6–9 years age. Stress.

[67] Buss C, Entringer S, Swanson JM, Wadhwa PD (2012). The Role of Stress in Brain Development: The Gestational Environment’s Long-Term Effects on the Brain. Cerebrum: the Dana Forum on Brain Science.

[68] Gitau R, Fisk NM, Teixeira JMA, Cameron A, Glover V (2001). Fetal Hypothalamic-Pituitary-Adrenal Stress Responses to Invasive Procedures Are Independent of Maternal Responses1. The Journal of Clinical Endocrinology & Metabolism.

[69] Burton GJ (2014). Optimising sample collection for placental research. Placenta.

[70] World Health Organisation. Low Birthweight, http://whqlibdoc.who.int/publications/2004/9280638327.pdf?ua=1 (2004).

[71] Navaratnam K (2013). A multi-centre phase IIa clinical study of predictive testing for preeclampsia: improved pregnancy outcomes via early detection (IMPROvED). BMC Pregnancy Childbirth.

[72] Livak KJ, Schmittgen TD (2002). Analysis of relative gene expression data using real-time quantitative PCR and the 2(-Delta Delta C(T)) Method. Methods.

