# Supplementary material for: Placental FKBP51 mediates a link between second trimester maternal anxiety and birthweight in female infants
Source: Sci Rep. 2018 Oct 11;8:15151. doi: 10.1038/s41598-018-33357-3 (PMC6181924; doi:10.1038/s41598-018-33357-3)
Supplement: Supplementary file 1 — Supplementary Information [file 41598_2018_33357_MOESM1_ESM.pdf]

Placental FKBP51 mediates a link between second trimester maternal anxiety and birthweight in female infants

Katie L. Togher [1, 2, 3, 4] (katie.togher@ucc.ie)

Gerard W. O'Keeffe [1, 2, 4], (g.okeeffe@ucc.ie)

Ali S. Khashan[1, 5], (a.khashan@ucc.ie)

Gerard Clarke[1, 2, 6], (g.clarke@ucc.ie) *

Louise C. Kenny[1, 3] (l.kenny@ucc.ie) *

[1] The Irish Centre for Fetal and Neonatal Translational Research (INFANT), Cork University Maternity Hospital and University College Cork, Cork, Ireland.

[2] APC Microbiome Institute, University College Cork, Cork, Ireland.

[3] Department of Obstetrics and Gynaecology, Cork University Maternity Hospital, University College Cork, Ireland.

[4] Department of Anatomy and Neuroscience, Western Gateway Building, University College Cork, Cork, Ireland.

[5] School of Public Health, Western Gateway Building, University College Cork, Cork, Ireland.

[6] Department of Psychiatry, Cork University Hospital and University College Cork, Cork, Ireland.

Supplementary Figures and Tables:

Supplementary Figure S1: *Flowchart of participants*

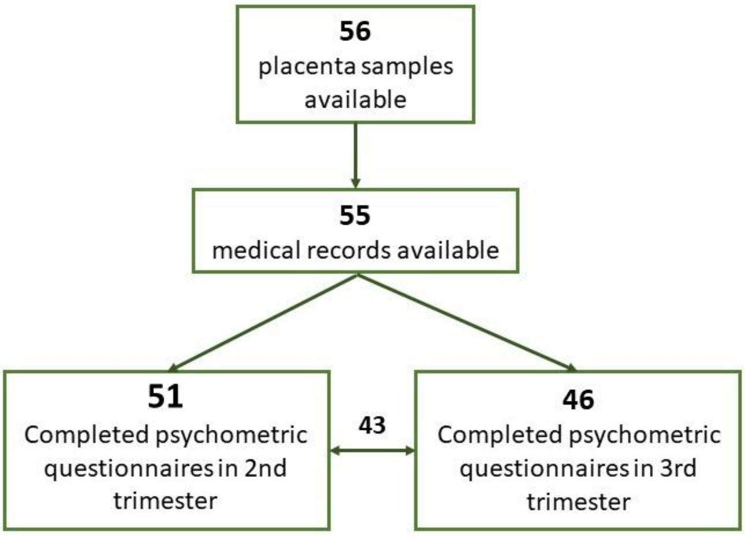

Supplementary Figure S1: Flowchart of participants included in this study. Placental samples were available from 56 pregnancies. Detailed medical records were available for 55 of these participants. 51 participants completed the Perceived Stress Scale (PSS), State Trait Anxiety inventory (STAI) and Edinburgh Postnatal Depression Scale (EPDS) in the second trimester. 46 participants completed these questionnaires in the third trimester. 43 women had completed these questionnaires at both time points.

Supplementary Figure S2: Measurement of maternal psychological distress.

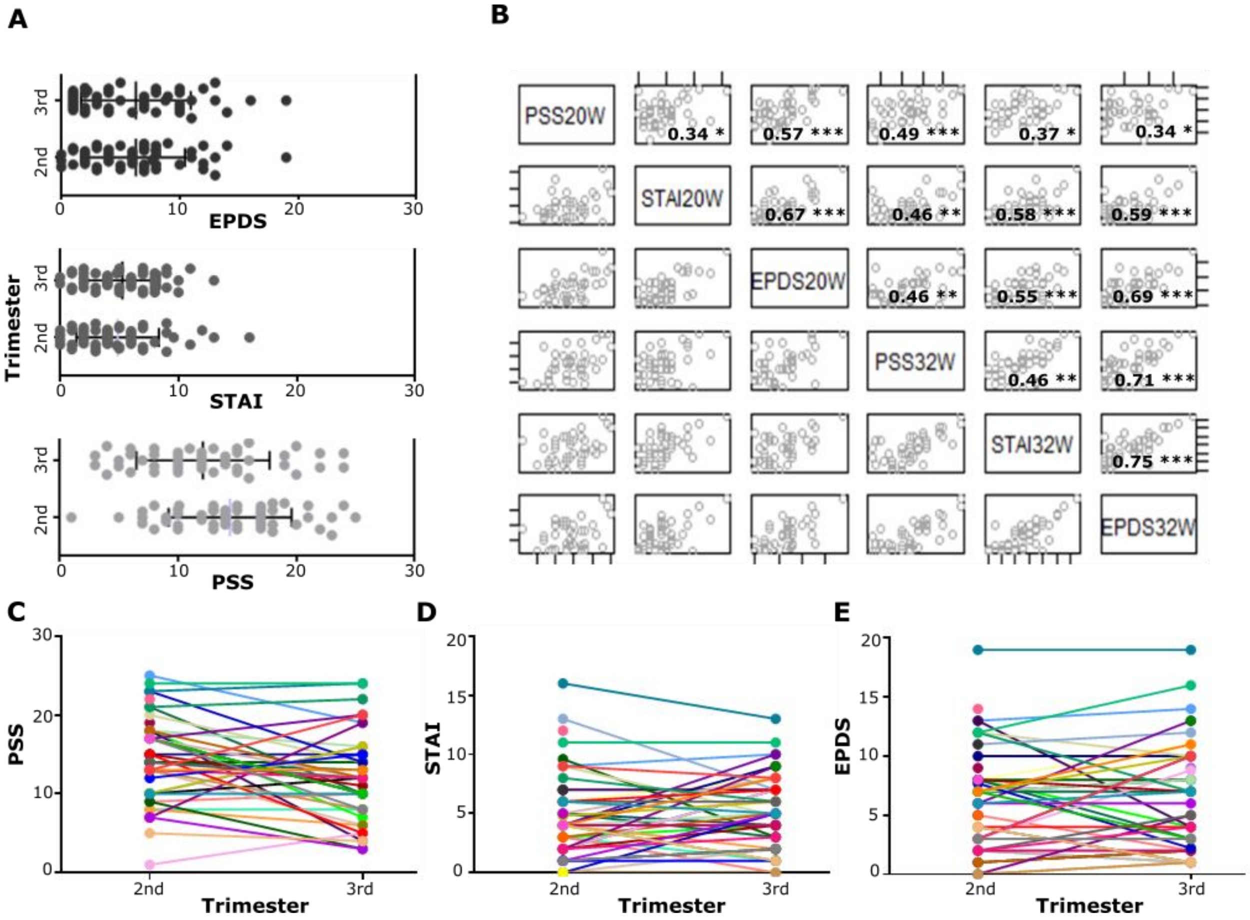

Supplementary Figure S2: Measurement of maternal psychological distress. (A) Distribution of questionnaire scores in the second and third trimester. Kolmogorov-Smirnov test for normality p>0.05. (B) Inter-correlation matrix of questionnaire scores. Spearman Rank Correlations p<0.05 *, p<0.01**, p<0.001 ***. No change observed in (C) PSS, (D) STAI and (E) EPDS scores from the second to third trimester. Paired Student's *t*-test, p>0.05 (n=43).

Supplementary Figure S3: Density plots of the distribution of outcome variables

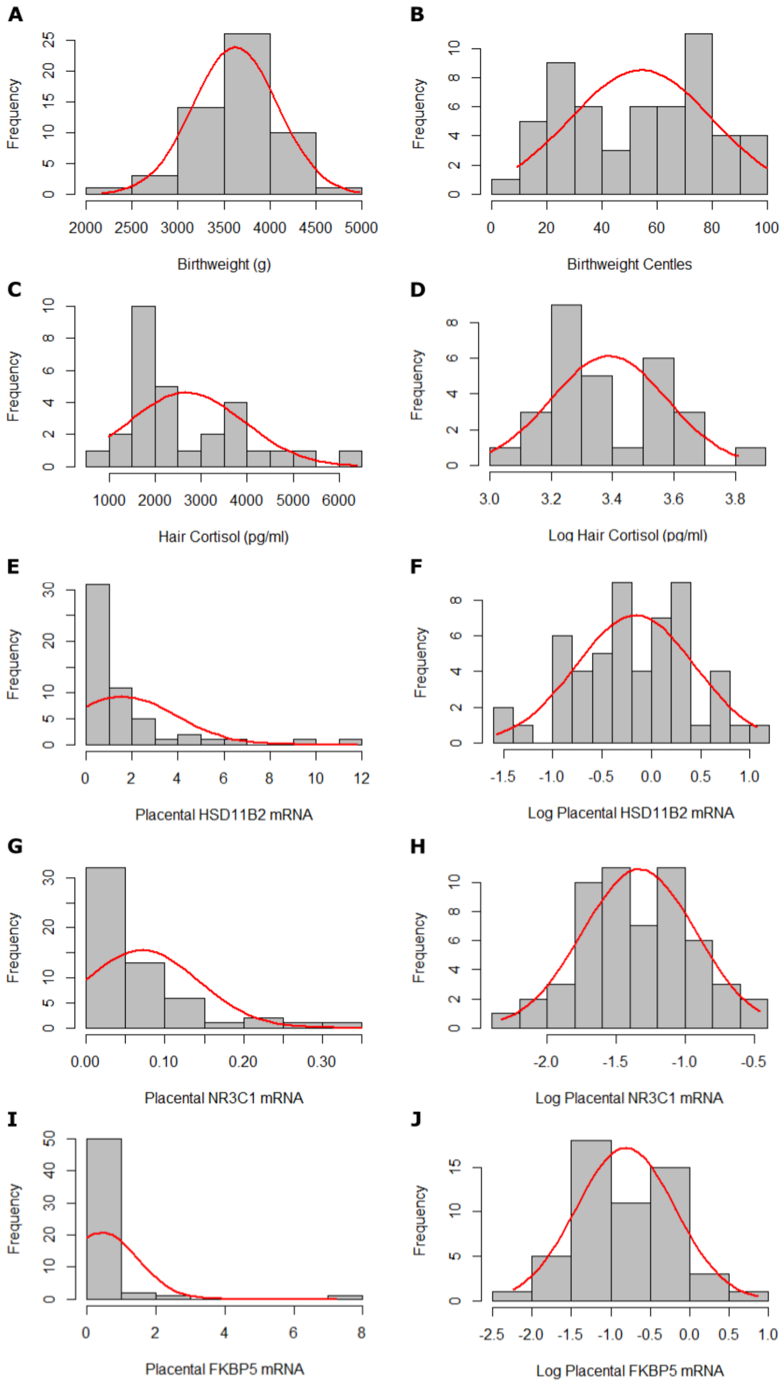

Supplementary Figure S3: Density plots of the distribution of outcome variables. (A) Birthweight and (B) birthweight centiles were normally distributed. (C, E, G, I) New-born hair cortisol levels and placental gene expression displayed a right skewed distribution. (D, F, H, J) The distribution of these outcomes was normalized following a log transformation.

Supplementary Table S1: Outcome and predictor variables with maternal age and BMI

	**Age**	**BMI**
PSS (2nd trimester)	β=-0.21, t_{50}=-1.52, p=0.134	β=-0.10, t_{50}=-0.77, p=0.445
STAI (2nd trimester)	β=-0.01, t_{50}=-0.09, p=0.926	β=0.13, t_{50}=0.91, p=0.370
EPDS (2nd trimester)	β=-0.02, t_{50}=-0.13, p=0.900	β=0.25, t_{50}=1.77, p=0.082
PSS (3rd trimester)	β=-0.03, t_{45}=-0.17, p=0.860	β=0.11, t_{45}=0.78, p=0.439
STAI (3rd trimester)	β=-0.09, t_{45}=-0.61, p=0.544	β=0.23, t_{45}=1.57, p=0.124
EPDS (3rd trimester)	β=0.01, t_{45}=0.07, p=0.945	β=0.27, t_{45}=1.88, p=0.066
Birthweight	β=-0.05, t_{54}=-0.38, p=0.707	**β=0.31, t_{54}=2.42, p=0.019**
Birthweight Centiles	β=0.09, t_{54}=0.68, p=0.503	**β=0.30, t_{54}=2.31, p=0.025**
Hair Cortisol	β=0.12, t_{28}=0.64, p=0.523	β=-0.06, t_{28}=-0.33, p=0.745
HSD11B2 mRNA	β=-0.17, t_{53}=-1.27, p=0.209	β=-0.09, t_{53}=-0.69, p=0.490
NR3C1mRNA	β=-0.24, t_{54}=-1.86, p=0.069	**β=-0.9, t_{54}=-3.09, p=0.003**
FKBP5 mRNA	β=0.05, t_{53}=0.41, p=0.681	β=0.05, t_{53}=0.37, p=0.711

Supplementary Table S1: Relationship between maternal stress predictor variables and outcome variables with maternal age and BMI. Linear regression analysis. Standardized beta coefficient (β). Maternal age and BMI were not correlated with maternal stress, hair cortisol levels or placental HSD11B2 and NR3C1 expression, $p > 0.05$. There was a positive correlation between birthweight, birthweight centiles and maternal BMI, $p < 0.05$. A strong negative correlation was observed between maternal BMI and placental NR3C1 expression $p < 0.01$.

Supplementary Table S2: Maternal stress and gestational age at delivery

Gestational Age			
	**Both**	**Males**	**Females**
**PSS (2nd trimester)**	β=-.18, t_{50}=-1.31, p=0.19	β=-.18, t_{23}=-0.90, p=0.37	β=-.16, t_{26}=-0.16, p=0.41
**PSS (3rd trimester)**	β=-.15, t_{45}=-1.00, p=0.32	β= -.18, t_{20}=-0.82, p=0.42	β=-.23, t26=-0.85, p=0.39
**STAI (2nd trimester)**	β=-.13, t_{50}=-0.91, p=0.36	β=.06, t_{23}=0.30, p=0.764	β=-.23, t_{26}=-1.22, p=0.23
**STAI (3rd trimester)**	**β=-.29, t_{45}=-2.02, p=0.04**	β=-.13, t_{20}=-0.59, p=0.56	β=*-.36, t_{24}=-1.88, p=0.07*
**EPDS (2nd trimester)**	β=-.20, t_{50}=-1.46, p=0.15	β=-.13, t_{23}=-0.65, p=0.51	β=-.20, t_{26}=-1.04, p=0.30
**EPDS (3rd trimester)**	β=-16, t_{45}=-1.13, p=0.26	β=-.22, t_{20}=-0.99, p=0.33	β=-.18, t_{24}=-0.90, p=0.37

Supplementary Table S2: Relationship between maternal stress and gestational age. Linear regression analysis. Standardized beta coefficient (β).

Supplementary Table S3: Dataset

Study_ID	Survey_Complete_3rd_Tri	Survey_Complete_2nd_Tri	BMI_1st_Tri	Maternal_Age	Gestational_Age_Delivery	Infant_Sex	Birth_weight	Birth_weight_Centiles	Infant_Cortisol	PSS_2nd_Tri	STAI_2nd_Tri	EPDS_2nd_Tri	PSS_3rd_Tri	STAI_3rd_Tri	EPDS_3rd_Tri	HSD11B2	NR3C1	FKBP5
P1	32	20	22.8	37	40	Female	3620	68	1901.45	9	3	5	10	0	2	4	0.02	0.32
P2	32	21	23.2	31	42	Female	4260	83	999	8	4	0	6	2	1	0.27	0.04	0.42
P3	32	20	31.2	32	40	Female	3660	54	999	12	6	8	15	8	11	1.72	0.02	0.05
P4	32	21	19.6	20	40	Male	3180	19	3482.99	18	3	8	7	4	4	1.33	0.08	0.09
P5	32	20	24.9	27	41	Male	3900	63	999	8	3	2	8	1	1	0.6	0.09	0.06
P6	33	20	28.1	23	40	Male	3760	54	1916.4	25	9	13	19	10	14	2.21	0.01	0.39
P7	999	20	26.1	37	39	Male	4100	82	3572.37	10	7	8	999	999	999	0.16	0.04	0.02
P8	33	999	23.7	35	39	Female	4130	97	6383.19	999	999	999	22	8	9	0.47	0.02	0.52
P9	999	19	19	31	40	Female	3090	27	999	17	4	8	999	999	999	1.44	0.07	0.17
P10	999	21	22.9	35	41	Female	3780	55	999	22	12	14	999	999	999	0.27	0.15	0.09
P11	34	21	22.4	28	41	Female	4180	78	1907.59	17	1	3	8	2	3	0.45	0.14	0.17
P12	31	20	22.5	31	40	Male	3700	47	999	5	4	4	4	1	1	0.18	0.08	0.67
P13	32	20	24.5	32	39	Male	3750	75	999	13	9	3	20	8	10	0.67	0.04	0.69
P14	32	19	25.5	31	39	Female	3750	78	999	13	4	7	13	8	11	0.59	999	0.09
P15	999	21	26.7	27	40	Female	4050	75	999	17	0	4	999	999	999	0.31	0.04	0.91
P16	34	20	29	27	40	Female	4400	92	2031.99	17	4	7	10	1	3	0.95	0.05	0.17
P17	32	21	20.7	19	39	Female	3000	28	1819.74	24	11	12	24	11	16	0.45	0.08	0.06
P18	32	21	26.3	27	41	Female	3620	47	1732.18	10	6	6	10	5	7	0.15	0.03	0.05
P19	32	19	24.3	33	40	Male	3700	62	3333.65	12	1	3	15	1	10	1.89	0.03	7.24
P20	32	20	39	39	39	Female	3850	79	4505.04	7	1	6	3	5	6	0.61	0.01	0.61
P21	999	20	22.1	30	40	Female	3610	56	999	13	5	6	999	999	999	0.03	0.06	0.05
P22	32	19	21.4	29	39	Female	2910	13	999	13	2	2	12	3	4	0.47	0.07	0.07
P23	34	21	23.9	33	37	Female	3150	72	999	14	6	2	12	6	5	2.54	0.02	999
P24	33	22	22.4	34	39	Female	3150	33	999	13	0	0	8	0	1	0.1	0.02	0.35

P25	36	20	23.5	31	40	Male	3800	56	2241.73	15	6	4	5	7	4	4.76	0.06	0.52
P26	999	19	26.9	26	40	Male	3570	40	999	7	3	5	999	999	999	0.98	0.01	0.42
P27	33	20	27.3	33	40	Female	3750	69	999	10.1	4.8	7	16	6	10	0.2	0.03	0.05
P28	33	999	23.1	41	39	Male	3200	28	1505.56	999	999	999	15	9	13	0.11	0.05	0.08
P29	31	20	25.6	40	39	Male	3200	20	1841.43	21	8	12	22	6	7	0.18	0.02	0.08
P30	32	21	38.5	35	37	Female	2910	37	1461.71	23	16	19	24	13	19	0.21	0.02	0.03
P31	999	21	32.7	31	40	Female	4980	100	1999.67	23	0	7.7	14	5	2.2	1.53	0.04	1.3
P32	35	20	22.8	33	40	Male	3470	28	3709.23	17	4	6	20	10	13	1.9	0.09	0.71
P33	32	20	24.2	30	41	Male	3100	9	999	7	2	0	19	9	5	0.13	0.12	0.05
P34	33	21	21	31	40	Male	3740	74	2452.89	17	4	2	10	4	2	1.44	0.23	0.05
P35	999	22	25.4	31	39	Female	3800	89	988.85	15	7	11	999	999	999	6.32	0.03	0.18
P36	34	19	31.4	31	40	Male	3520	34	3565.91	15	2	8	11	4	7	0.35	0.03	0.43
P37	33	22	28.4	31	40	Male	4160	78	999	1	2	3	5	7	8.8	0.54	0.09	0.12
P38	32	19	24.7	28	41	Male	3510	28	4164.34	18	3	1	12	5	2	0.13	0.03	0.02
P39	33	20	24.7	34	40	Male	4200	89	999	10	1	1	12	2	2	0.05	0.01	0.02
P40	32	21	26.4	31	41	Male	3780	40	2461.19	10	13	11	16	7	12	0.04	0.04	0.01
P41	34	999	23.5	30	40	Female	3720	78.9	3771.62	999	999	999	8	2	1	1.97	0.2	0.77
P42	33	999	24.3	38	34	Female	2170	24	999	999	999	999	6	6	1	0.31	0.03	0.04
P43	32	20	22.2	31	40	Female	3820	73	1871.1	18	4	7	16	5	8	9.88	0.09	0.21
P44	32	22	23.1	32	41	Male	3460	26	999	20	9	12	14	4.8	10	1.02	0.15	0.02
P45	31	21	22.8	23	40	Female	3030	15	999	17	5	2	12	8	1	4.06	0.12	0.18
P46	32	21	20.3	34	39	Male	3340	31	2214.77	13	0	1	6	2	2	1.05	0.02	1.83
P47	32	20	29.1	34	39	Female	4080	95	2724.87	14	2	8	14	7	8	11.78	0.02	2.21
P48	32	21	28.7	33	41	Male	3550	30	1463.18	9	9.6	4	3	3	1	0.71	0.23	0.29
P49	999	999	999	999	999	999	999	999	999	999	999	999	999	999	999	999	0.08	999
P50	33	20	26.8	26	41	Female	3750	45	999	14	5	8	13	3	3	2.35	0.01	0.07
P51	34	21	20.8	35	40	Male	3100	13	5042.91	21	4	7	10	4	7	5.45	0.02	0.45
P52	32	21	22.4	30	40	Female	3880	79	999	17	5	7	10	4	8	2.23	0.09	0.06
P53	33	21	22.7	23	39	Female	3280	57	1813.69	15	4	10	15	9	10	1.26	0.35	0.25

P54	33	21	19.3	28	41	Female	3310	21	999	18	7	13	4	7	4	2.52	0.27	0.14
P55	999	19	32.3	24	40	Male	4110	69	999	14	6	5	999	999	999	999	0.03	0.22
P56	999	20	23.7	28	39	Male	3700	64	999	19	7	9	999	999	999	0.52	0.14	0.01

Supplementary Table S3: Raw dataset used to complete this work. Missing values (999). Abbreviations: Trimester (Tri). Placental gene expression normalised to GAPDH, data shown are delta-delta Ct values.
